# The prevalence and associated factors of success of labor induction in Hargeisa maternity hospitals, Hargeisa Somaliland 2022: a hospital-based cross-sectional study

**DOI:** 10.1186/s12884-023-05655-w

**Published:** 2023-06-13

**Authors:** Fatima Qasim Farah, Getie Lake Aynalem, Asmra Tesfahun Seyoum, Getachew Muluye Gedef

**Affiliations:** 1 Department of Midwifery, Bosaso University, Bosaso, Somalia; 2 School of Midwifery, College of Medicine and Health Science, University of Gondar, Gondar, Ethiopia

**Keywords:** Success of induction of labor, Associated factors, Hargeisa maternity hospital

## Abstract

**Background:**

The induction of labor is an artificial initiation of labor and has become one of the most common interventions in modern obstetrics to improve maternal and neonatal health. Understanding the prevalence and pregnancy outcomes following labor inductions is crucial in regions with high rates of maternal mortality and morbidity because of insufficient access to comprehensive emergency obstetric care. Therefore, this study aimed to assess the prevalence and associated factors of the success of induction of labor in Hargeisa maternity hospital Somaliland.

**Methods:**

A hospital-based cross-sectional study was employed among 453 women in Hargeisa maternity hospitals, Somaliland from January 1st to March 30th, 2022. Data were entered using Epi data version 4.6 and analyzed by using SPSS version 25. Bivariable and multivariable logistic regression were used to identify the associated factors with the success of labor induction and an odds ratio with a 95% confidence interval was used to measure the strength of the association. A P-value of ≤ 0.05 was considered statistically significant in multivariate analysis.

**Results:**

Of a total of 453 study participants who had undergone induction of labor, 349 (77%) of them had successful induction of labor with a 95% CI: 73%, 81%. Favorable Bishop score (AOR = 3.45, 95% CI: 1.98, 5.99), time from the start of induction to delivery < 12 h (AOR = 4.01, 95% CI: 2.16, 7.450), non-reassuring fetal heart rate pattern (AOR = 0.42, 95% CI: 0.22, 0.78) and amniotic fluid change to meconium (AOR = 0.43, 95% CI: 0.23, 0.79) were significantly associated with the success of labor induction.

**Conclusion:**

This study implies that three out of four women who underwent induction had successful induction of labor. Favorable bishop score, time from the start of induction to delivery < 12 h, non-reassuring fetal heart rate pattern, and amniotic fluid change to meconium were significantly associated with the success of labor induction. The hospital should establish a clear bishop scoring system and there should be a strict follow-up on the condition of the fetal heartbeat and take corrective actions as needed. The factors related to healthcare facilities and providers need to be addressed by additional prospective studies.

## Introduction

Induction of labor (IOL) is the artificial initiation of labor after the age of fetal viability and before the onset of spontaneous labor to achieve a vaginal delivery when the benefits of delivery outweigh the risks of continuing the pregnancy [1–3]. The common indication for IOL includes maternal, fetal, social, or a combination of these factors [2].

Induction of labor can use a variety of methods, pharmacological and non-pharmacological [1, 4]. Cervical ripening agents, artificial rupture of membranes, and uterine stimulation with oxytocin are all methods for inducing labor [5].

Rates of IOL vary from region to region [2], with the progressive increase nearly doubling the incidence in some developed countries [6]. According to the World Health Organization (WHO), IOL may account for up to 25% of all term deliveries in developed countries and account for only 4.4% of deliveries on average in African countries [3, 7]. African rates of induction labor are still very far from the expected, averaging 60–80.2% unmet need for labor induction [8, 9].

Africa has the highest maternal mortality as well as the highest stillbirth [10]. The estimated maternal mortality ranks Somaliland as having the world’s 5th highest level of maternal mortality, approximately 1,300 maternal deaths occur annually and 42 neonatal death per 1000 live births occur in Somaliland [11].

Improving care for women during labor is a necessary step forward to achieve health targets and sustainable development goals. IOL has directly relevant to reducing maternal and neonatal mortality as it has the potential for preventing maternal complications and improving pregnancy outcomes [6].

Despite the fact that IOL is an essential practice in preventing neonatal and maternal mortality and morbidity, it is not always successful and can have unfavorable results. A review of the literature showed that, among women who had IOL, only 84% of mothers in Saudi Arabia [12], 80.6% in India [13], 75.9% in Nigeria [14], and 59.7% in Ethiopia [5] achieved a vaginal delivery. Maternal age [15, 16], place of residence [17], body mass index (BMI) [12, 18], Parity [12, 18], [17, 19, 20], gestational age (GA) [12, 17, 18], Bishop score [12, 18–21], APGAR score [12, 22], fetal heart rate pattern [17], indications [13, 18, 23], hypertensive disorder[24] and methods of induction [17, 24, 25] were factors associated with the success of labor induction.

When induction is not successful, the mode of delivery is the caesarian section which could be associated with a higher rate of excessive blood loss, puerperal sepsis, and maternal mortality. For the IOL to succeed, it is crucial to detect and improve the gap. However, there haven’t been sufficient studies conducted and none have been done in the study area.

In an area with a high rate of maternal mortality and morbidity due to poor access to comprehensive emergency obstetric care, knowing the prevalence, indications, and pregnancy outcomes following inductions of labor is crucial. Therefore, this study aimed to determine the prevalence of successful IOL and associated factors among mothers who had just given birth in the maternity hospitals of Hargeisa, Somaliland. As a result, the findings from this research could make it possible for women who require induction labor to get improved quality of care in hospitals.

**Fig. 1:**
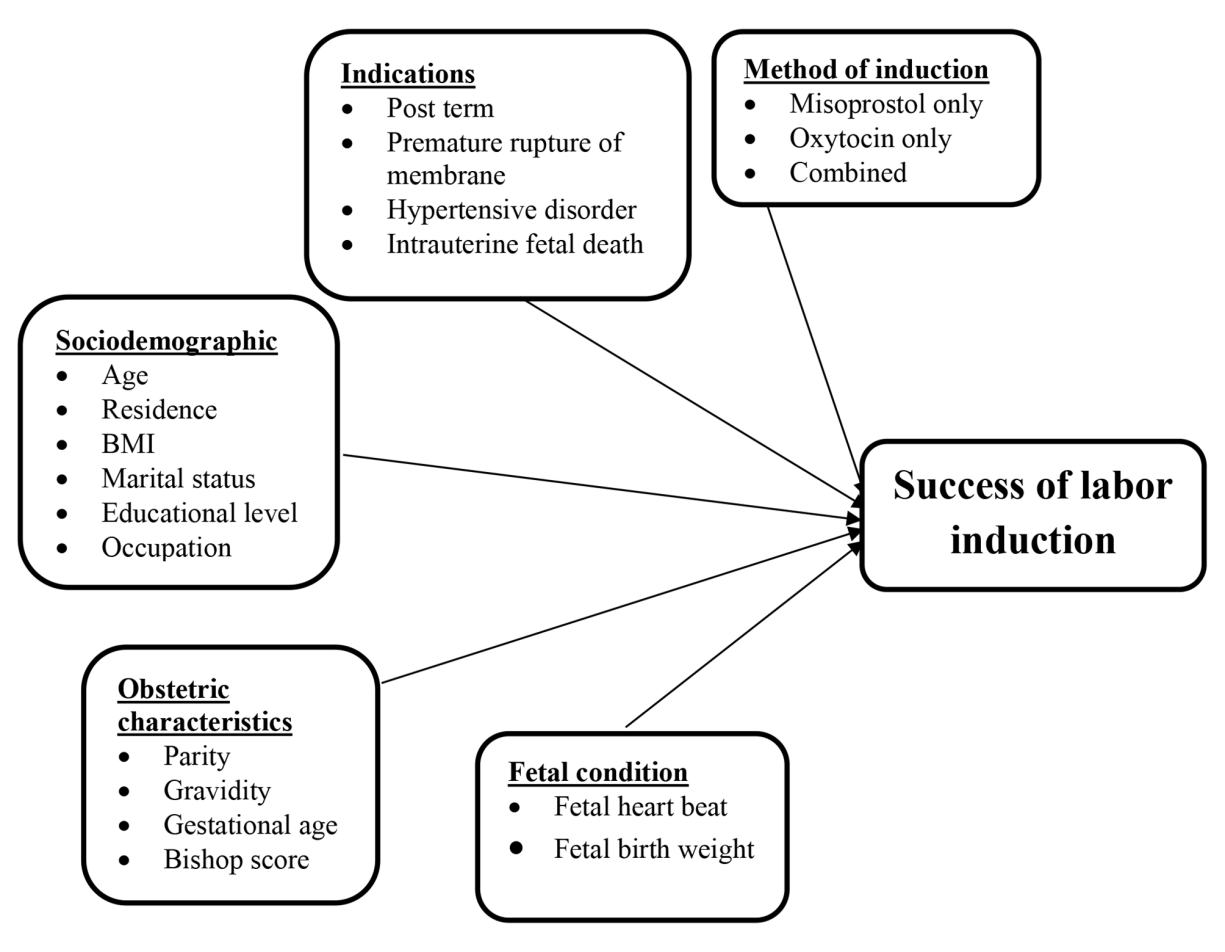
Conceptual framework for associated factors of success of labor induction

Conceptual framework (Fig. 1).

## Methods

### Study design, setting and period

A hospital-based cross-sectional study was employed from January 1st to March 30th, 2022 in Hargeisa maternity hospitals, Hargeisa Somaliland, the capital city of Somaliland. In Hargeisa, there are four maternity hospitals which are Hargeisa group hospital, Kaah community hospital, Edna maternity hospital, and Gargaar hospital. Hargeisa group hospital is the oldest and largest and opened in 1953. Kaah community hospital and Edna maternity hospital are the second largest and they started providing the service to mothers in 1991 and 1998, respectively. Gargaar hospital has been giving to mothers for more than ten years.

### Study population and the eligibility criteria

Study population: The study population was all immediate postpartum mothers who underwent induction in Hargeisa maternity hospitals during the data collection period.

Inclusion criteria: All immediate postpartum mothers who had undergone IOL at Hargeisa maternity hospitals during the study period were included in the study.

Exclusion criteria: Mothers who couldn’t respond during the interview due to severe acute mental illness and unwillingness to participate were excluded from the study.

### Sample size determination and sampling procedure

The primary and secondary objectives were comparably considered for the sample size calculation.

Based on the first objective, the sample size was determined by using a single population proportion formula, n=(Zα/2)^2^P(1 − P)/d^2^ with the following assumptions. Using Proportion (p) = 63.2% from a previous study conducted in Jigjiga Ethiopia [26], a 95% confidence interval (CI), a 5% margin of error and by considering a 10% non-response rate, the final sample size was 393.

The sample size for the second objective (associated factors) was done by using EPI INFO version 7.1 statistical software based on the double population proportion formula. The four factors (residence, post-term, parity, and fetal heart rate pattern) which had a strong association with the success of IOL were found in three studies done in Ethiopia [17, 20, 27]; we then selected the variable (post-term) that provided the highest sample size, which is 412; by including a 10% non-response rate, the final sample size for this study was 453.

**Fig. 2:**
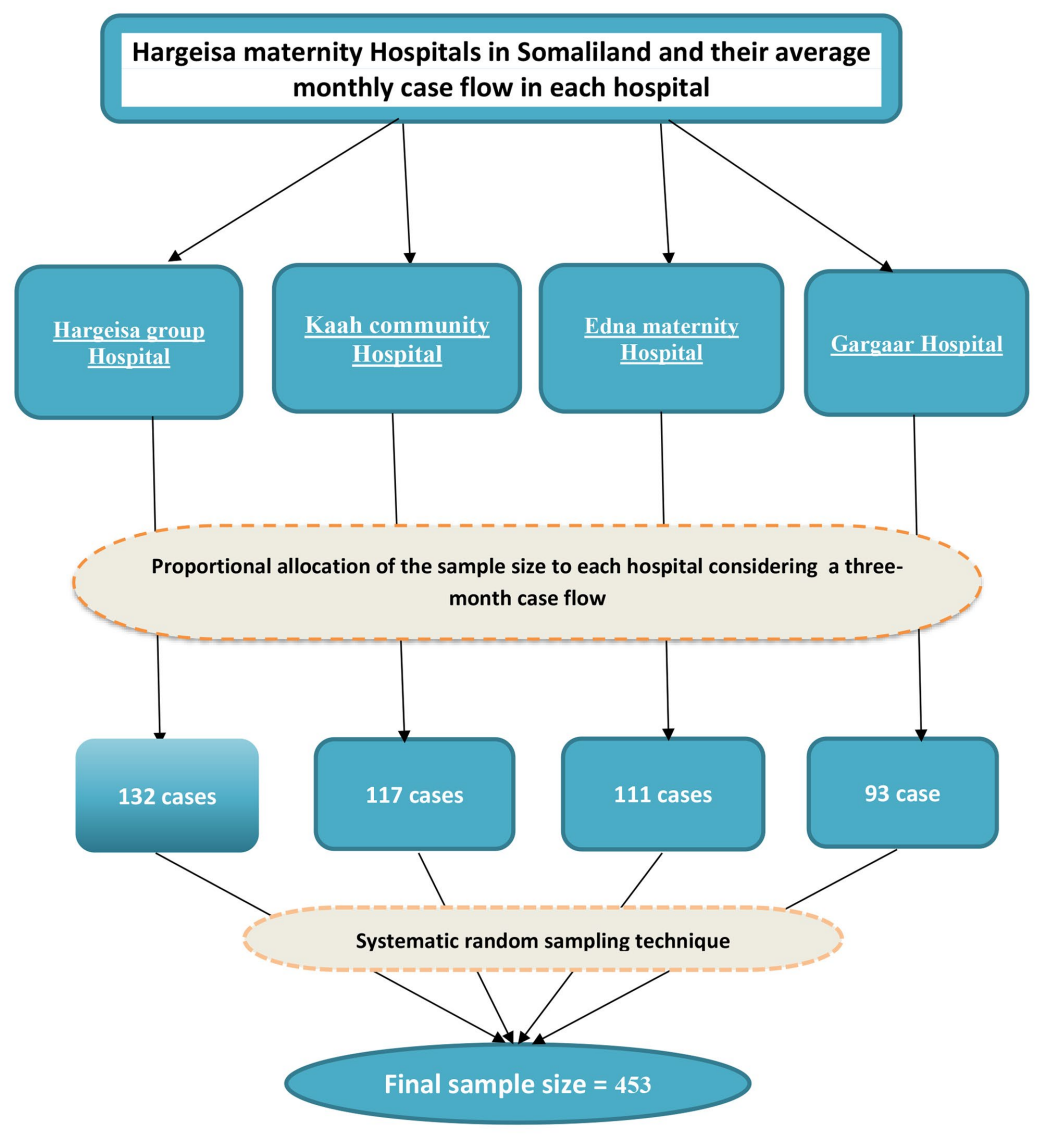
Schematic representation of sampling procedure for the study to assess the prevalence and associated factors of success of labor induction in Hargeisa maternity hospitals, Hargeisa Somaliland 2022

All four maternity hospitals in Hargeisa Somaliland were included in the study. In four hospitals, there were about 225 pregnant women (on average) admitted for labor induction each month. The required sample size from each hospital was obtained by allocating proportionally based on their average monthly case flow in a three-month duration (Hargeisa group Hospital = 132, Kaah community Hospital = 117, Edna maternity Hospital = 111 and Gargaar Hospital = 93). A total of 453 mothers admitted for induction labor were selected using a systematic random sampling technique (Fig. 2).

### Data collection tools and data quality control

The data were collected prospectively by face-to-face interviews and the client’s medical record review technique using pretested structured and interviewer-administered questioners and checklist and anthropometry measurement. The questionnaire was adapted from the WHO global survey for maternal and perinatal health and some modifications was made to incorporate all objectives [22, 24, 28]. The questionnaire contains the socio-demographic factors and obstetric factors. Four BSc nurses working in the study hospitals in another ward collected the data from January 1st to March 30th, 2022 and were supervised by two BSc midwives and the investigator.

We initially adapted the questionnaire in English, then translated it into Somali which is the local language of the study area. Then it was translated back to English by language experts to see its consistency. To see the appropriateness of the tool and to ensure the quality of data gathered, we made a pre-test on 5% (23 participants) of the sample size at the Jigjiga referral hospital before the actual data collection started. We made the necessary modifications to the questionnaires based on the identified gaps. We gave a one-day training for data collectors and supervisors on the objective and relevance of the study, data collection techniques and procedures before the actual data collection. The supervisors and principal investigators reviewed and checked for completeness of the questionnaire every day after data collection. Principal investigators calibrated anthropometry measurements every week by triangulating them with standard weight.

### Data management and statistical analysis

After we checked the data for its completeness and consistency, data were cleaned, coded, and entered into Epi data version 4.6 and exported to the Statistical Package for Social Sciences (SPSS) version 26 software package for data analysis. We used bivariable and multivariable analysis to assess the association of different potential factors with the outcome variable. Variables with a p-value of 0.2 were candidates for multivariable logistic regression to control the confounding effect and in multivariable logistic regression variables with a p-value of ≤ 0.05 were taken as statistically significant determinants of the success of IOL. In addition, we used Hosmer Lemeshow goodness-of-fit test to check model fitness with a p-value > 0.05. Multicollinearity was checked using the Variance inflation factor (VIF), in which a VIF of less than ten was considered to be acceptable.

### Study variables

The dependent variable was the success of IOL whereas the independent variables were the sociodemographic factors (age, place of residence, family income, body mass index), obstetric characteristics (parity, gravidity, gestational age, bishop score), indications of IOL (premature rupture of membrane (PROM), post-term pregnancy, intrauterine fetal death (IUFD), hypertensive disorder, Oligohydramnios, and intrauterine growth restriction (IUGR), methods of induction (misoprostol only, oxytocin only, and combined) and fetal condition (fetal weight, and fetal heart rate pattern).

### Definition of terms

#### Induction of labor

is the artificial initiation of labor to deliver the fetus vaginally after the fetus has reached viability (after the 28 weeks of gestation) and before the onset of spontaneous labor [24].

#### Successful induction of labor

when a woman had achieved vaginal birth after labor was induced [24].

#### Body mass index (BMI)

Is defined as the weight in kilograms divided by the square of the height in meters(kg/m)^2^ and is commonly used to classify underweight (BMI < 18.5), normal weight (BMI = 18.5–2.9), overweight (BMI ≥ 25-29.5) and obese (BMI > 30) in adults [29]. Maternal BMI was calculated based on maternal height and weight measurements provided at admission.

#### Bad obstetrics history

previous unfavorable fetal and maternal outcomes.

#### Fetal distress

Normal fetal heart rate pattern was defined as a baseline heart rate of 110–160 beats per minute with variability of 5–25 beats per minute and no repetitive decelerations. All findings which deviated from this normal fetal heart rate pattern definition were considered fetal distress [20].

#### APGAR score

low if the five-minute APGAR score is less than seven [24].

#### Bishop score

a score of five or lower represents an unfavorable cervix, whereas a score of greater than five indicates a ripened cervix [20].

#### Premature rupture of membranes

the rupture of membranes before the onset of labor [24].

## Results

### Sociodemographic characteristics

A total of 453 study participants were interviewed, yielding a 100% response rate. The mean age of the study participants was 29.17 years with a standard deviation (SD) of 6.14, and nearly six in ten 262 (57.8%) respondents were in the age group of 25–35 years. Nearly six in ten 266 (58.7%) study participants were urban residents (Table 1).

**Table 1:** socio-demographic characteristics of study participants in Hargeisa maternity hospital, Hargeisa, Somaliland, 2022 (n = 453)

Variable	Category	Frequency	Percentage
Age	< 20	20	4.4
	20–34	343	75.7
	≥ 35	90	19.9
Residence	Urban	266	58.7
	Rural	187	41.3
Family average monthly income	≤ 100$	168	37.1
	101–399$	219	48.3
	≥ 400$	66	14.6
Body mass index	Underweight	80	17.7
	Normal weight	261	57.6
	Overweight	105	23.2
	Obese	7	1.5

### Obstetric characteristics of the study participants and the prevalence of success of labor induction

Of the total study participants, 325 (71.1%) were multiparous. More than half, 249 (55%) of the study participants’ gestational age was from 37 to 41 weeks and SD of 4.17. More than three-quarters (77%) of study participants had given birth vaginally (successful IOL) with a 95% confidence interval of 73%, 81% (Table 2).

**Table 2:** Obstetric characteristics of study participants in Hargeisa maternity hospital, Hargeisa, Somaliland 2022 (n = 453)

Variables	Category	Frequency	Percentage
Gravidity	Primigravida	98	21.6
	Multigravida	355	78.4
Parity	Primi para	128	28.3
	Multipara	325	71.7
Bad obstetric history	Yes	81	17.9
	No	372	82.1
Gestational age (Weeks)	28–36	137	30.2
	37–41	249	55.0
	≥ 42	67	14.8
Mode of delivery	Vaginally	349	77.0
	Cesarean section	104	23.0
Feta birth weight (gram)	< 2500	103	22.7
	2500–3999	251	55.4
	≥ 4000	99	21.9

### Indication and method of induction of labor

In nearly two-thirds of 292 (64.5%) study participants’ the method of induction was misoprostol only. About three-quarters of study participants, 346 (76.4%), gave birth within 12 h of the start of induction labor. Regarding the indication of induction, there were different reasons for induction of labor, and PROM takes the large share that accounts for 28.7%, followed by IUFD (26.3%) and hypertensive disorder (23%) (Table 3).

**Table 3:** The indication and prevalence of success of induction of labor in Hargeisa maternity hospital, Hargeisa, Somaliland 2022 (n = 453)

Variables	Category	Frequency	Percentage
Type of induction	Planned	252	55.6
	Emergency	201	44.4
Indication for induction	PROM	130	28.7
	IUFD	119	26.3
	Hypertensive disorder	104	23
	Post-term	84	18.5
	Others^**a**^	16	3.5
Method of induction	Oxytocin only	112	24.7
	Misoprostol only	292	64.5
	Combined	49	10.8
The membrane ruptured before induction	Yes	157	34.7
	No	296	65.3
Bishop score	Favorable	296	65.3
	Unfavorable	157	34.7
Time from start of induction to delivery	< 12 h	346	76.4
	≥ 12 h	107	23.6
Uterine hyperstimulation	Yes	50	11.0
	No	403	89.0
NRFHRP following induction	Yes	107	23.6
	No	346	76.4
Amniotic fluid changes to meconium	Yes	136	30.0
	No	317	70.0

^**a**^Oligohydramnios, Diabetes mellitus, and Intrauterine growth restriction; Congenital anomalies;

PROM: Premature Rupture of Membrane; IUFD: Intrauterine Fetal Death; NRFHRP: Non-reassuring Fetal Heart Rate Pattern

### Factors associated with the success of labor induction

In this study, the association of different factors with the success of induction was assessed by using bivariable and multivariable logistic regression analysis. On the crude analysis, parity, gestational age, fetal weight, indication for induction, bishop score, uterine hyperstimulation, non-reassuring fetal heart rate pattern, change in the color of amniotic fluid and the start of induction to delivery time were factors associated with the success of IOL. Variables with p-values of less than 0.2 in the bivariable analysis were entered into the multivariable analysis. After adjusting for possible confounding factors in the multivariable analysis only bishop score, time from the start of induction to delivery, non-reassuring fetal heartbeat pattern, and changes in the color of amniotic fluid had a significant association with the success of labor induction.

The odds of successful IOL were 3.45 times (AOR = 3.45, 95% CI: 1.98, 5.99) more likely in favorable bishop score than unfavorable. Similarly, the odds of IOL were higher among women who gave birth within 12 h of the start of labor induction compared with their counterparts (AOR = 4.01, 95% CI: 2.16, 7.450).

On the other hand, the success of IOL was 59% times lower among women with NRFHRP (AOR = 0.42, 95% CI: 0.22, 0.78). Furthermore, the success of IOL was 57% times less likely among women with a change in the color of amniotic fluid (AOR = 0.43, 95% CI: 0.23, 0.79) (Table 4).

**Table 4:** The bivariate and multivariate analysis of factors associated with successful induction of labor in Hargeisa maternity hospital, Hargeisa, Somaliland, 2022 (n = 453)

Variables	Success of induction		COR (95% CI)	AOR (95%CI)	p-value
	**Yes n(%)**	**No n(%)**			
Parity					
Primipara	77 (58)	51 (39.8)	1	1	
Multipara	272 (83.7)	53 (16.3)	3.39 (2.15, 5.39)	1.73 (0.96–3.10)	0.066
Gestational age (Weeks)					
28–36	125 (91)	12 (9)	**2.76 (1.41, 5.41)** ^*^	1.22(0.44, 3.38)	0.702
37–41	185 (74)	64 (26)	**1.88 (1.05, 3.36)** ^*^	1.04 (0.41, 2.63)	0.927
≥ 42	39 (58)	28 (42)	1	1	
Fetal weight (gram) < 2500 2500–3999 > 4000	73 (70.9) 195 (77.7) 81 (81.8)	30 (29.1) 56 (22.3) 18 (18.2)	**0.54(0.28, 0.05)** ^*^ 0.77 (0.43, 0.39) 1	0.58(0.26,1.30) 0.81(0.40,1.66) 1	0.192 0.580
Indication of induction Post-term PROM IUFD Hypertensive disorder Others^a^	51 (61) 95 (73) 115 (97) 69 (66) 12 (75)	33 (39) 35 (27) 4 (3) 35 (34) 4 (25)	**0.39 (0.10,1.49)** ^*^ 0.67 (0.18, 2.52) **5.26(1.13, 24.59)** ^*^ 0.52(0.14, 1.95) 1	0.61 (0.12, 2.96) 0.81 (0.19, 3.52) 4.42 (0.8, 24.48) 0.77 (0.17, 3.36) 1	0.541 0.781 0.088 0.725
Bishop score Favorable Unfavorable	248 (84) 101 (64)	48 (16) 56 (36)	2.86 (1.82, 4.49) 1	3.45 (1.98, 5.99) 1	**0.001**
Uterine hyperstimulation					
Yes	46 (92)	4 (8)	3.79(1.33, 10.80)	2.96 (0.92, 9.51)	0.067
No	303 (75)	100 (25)	1	1	
NRFHB					
Yes	64 (60)	43 (40)	0.32 (0.19, 0.51)	0.42 (0.22, 0.77)	**0.007**
No	285 (82)	61 (18)	1	1	
Change in the color of amniotic fluid					
Yes	81 (60)	55 (40)	0.24 (0.2,0.4)	0.43 (0.23, 0.79)	**0.007**
No	272 (86)	45 (14)	1	1	
Time from start of induction to delivery					
< 12 h	289 (84)	57 (17)	3.97 (2.45, 6.39)	4.01 (2.16, 7.46)	**0.001**
≥ 12 h	60 (56)	47 (44)	1	1	

IUFD: Intrauterine uterine fetal death; NRFHB: Non-reassuring fetal heartbeat pattern; PROM: Prolonged Rupture of Membrane

^**a**^Oligohydramnios, Diabetes mellitus, Intrauterine growth restriction, Congenital anomaly

^*^Catagories that were associated with the success of induction of labor in a bivariate analysis

## Discussion

Although IOL has become one of the most common daily practices in modern obstetrics there is a limitation in undertaking a study on the prevalence, outcomes and associated factors of success of labor induction and most of the studies concentrated on the failure rate of labor induction as a sole outcome. As a result, we conducted this study to assess the prevalence and associated factors of success of labor induction in Haregisa maternity Hospital, Somaliland.

In this study out of a total of 453 women who underwent IOL, 349 (77%) mothers had successful IOL, with a 95% confidence interval of 73%, 81%. This result is comparable with a study conducted in public hospitals of Mekelle town (76%) [30]. The use of oral misoprostol as one method of induction in both studies could be the cause of the similarities.

However, this rate is slightly higher than the studies conducted in Addis Ababa (59.7%) [22], and Debre Berhan (65.9%) [24]. This discrepancy could be due to the difference in a hospital setup, skilled professionals, and availability of a different method of IOL. This rate varied little per the country or the method used.

Furthermore, the current study finding is lower than a study conducted in Dire Dawa (83.6%) [17]. This discrepancy could be the result of differences in the selection criteria used in earlier studies which defined unsuccessful induction only if mothers failed to attain an active first stage of labor after six to eight hours. In this study, any labor that resulted in a cesarean section following labor induction was considered unsuccessful induction regardless of the duration.

The current study showed a significant association between a women’s bishop score and the success of induction of labor, women with a favorable bishop score were 3.45 times more likely to have successful induction than unfavorable bishop scores. This is consistent with a study done in southwest Ethiopia which found that women who had a bishop score of more than five were four times more successful than compared with a Bishop Score of less than five [31]. this is similar to the study done in Addis Ababa army referral hospitals in Ethiopia the odds of successful induction being seven times more likely in women with favorable Bishop Score than unfavorable [22]. In another study conducted in southeast Ethiopia, those with unfavorable bishop scores were five times more likely to have failed IOL [28]. The condition of the cervix at the start of induction is an important predictor/ crucial factor, if the cervix is not ripe, induction of labor results in a high failure rate.

The success of induction was four times more likely if the time from the start of IOL to the delivery of the baby is less than 12 h. Contrarily, a study conducted in the Arsi zone in southeast Ethiopia showed that the time from the start of induction to delivery of the baby of fewer than 12 h was more likely to fail IOL than the start of labor to delivery of a baby 12 h or more [28]. This difference might be because more than three-quarters of our study participants were multigravida cervical dilatation faster than primigravida mothers.

The success of induction of labor was 59% lower among women with non-reassuring fetal heart rate patterns than regular heart rate patterns. This finding is in line with a study conducted in Dire Dawa that found the success of labor induction was 90% less likely in respondents with non-reassuring fetal heart rate patterns [17]. In a similar study, women with a non-reassuring fetal heartbeat at the beginning of induction were 0.36 times less likely to have successful induction labor [22]. This could be because the presence of fetal heart rate abnormality could cause fetal distress in the fetus and guide to fetal death and failure of induction.

Regarding the change of amniotic fluid, the success of induction labor was 57% times less likely among women with a change in the color of amniotic fluid. Similarly, another study in India indicated a significant association between amniotic fluid-stained meconium and failed induction [32]. And this might be due to that pregnancy-induced hypertension predisposing to fetal compromise as a result of uteroplacental insufficiency. In addition, it may necessitate preterm labor induction before the maturation of the fetus and may easily lead to a non-reassuring fetal heartbeat pattern when uterotonic agents are administered.

In this study, PROM was the most common indication for labor induction, accounting for 28.7% of cases, followed by IUFD and hypertensive disorder (23%). This is consistent with a study conducted in Mekelle’s public hospitals [30] and Egypt [29] which found that PROM was the most frequent indication of labor induction. Moreover, misoprostol (64.5%) and IV oxytocin infusions (24.7%) were the two techniques most frequently utilized in this study to induce labor. This is in line with a previous study that revealed the most widely used methods of labor induction were misoprostol and intravenous oxytocin infusion[29]. The choice of whether to induce labor with oxytocin or misoprostol depends on the state of the cervix, the availability of misoprostol and the protocol applied within the particular health facility.

Previous research found associations between maternal BMI, parity, and fetal weight with the success of labor induction. However, none of them were significant variables in the current study.

### The strengths and limitations of the study

The results of the study would provide insight on the outcomes of a common obstetrical intervention, which is IOL in low-resource settings for further improvement of the quality of care. However, this study’s design, which can only explain the existence of a relationship between variables rather than a causation-effect relationship, and its retrospective nature, which raises the possibility of recall bias, are some of its limitations. Also, this study did not address factors related to the healthcare system and policies.

## Conclusion

The study revealed that three out of four women who had undergone induction labor had successful induction. Factors such as Bishop score, non-reassuring fetal heart rate pattern, change in color of amniotic fluid, and time from the start of IOL to delivery had a significant association with the success of induced labor. The hospital should set up a clear bishop scoring system and the health care provider should assess the Bishop Score before the woman undergoes induction and follow the fetal heartbeat strictly and act accordingly to achieve a desirable outcome of induction of labor. To achieve the desired outcome of labor induction, the use of surgical methods of induction (rupture of membrane) in conjunction with various labor induction methods should be tried. The minister of health should develop evidence-based clinical practice guidelines for the IOL and ensure its implementation. Further prospective research is needed to address health facility and healthcare providers’ related factors as well as to determine the cause of IUFD and what makes it prevalent in the research area.

## Data Availability

The data sets used and or analyzed during the current study are available from the corresponding author on reasonable request via email.

## Abbreviations

AOR: Adjusted odds ratio
COR: Crude odds ratio
APGAR: Appearance, Pulse, Grimace, Activity, Respiration
IOL: Induction of labor
IUFD: Intrauterine uterine fetal death
NRFHRP: Non-reassuring fetal heart rate pattern
PROM: Prolonged rupture of membrane
